# Genome‐Guided and De Novo Transcriptome Analysis of a Newly Isolated *Neopyropia yezoensis* Strain With Heat Tolerance

**DOI:** 10.1155/ijog/9130170

**Published:** 2025-12-08

**Authors:** Jong-il Choi, Jiae Kim

**Affiliations:** ^1^ Department of Biotechnology and Bioengineering, Chonnam National University, Gwangju, Republic of Korea, jnu.ac.kr

**Keywords:** heat tolerance, *Neopyropia yezoensis* (Daebudo), transcriptome

## Abstract

**Background:**

The escalating threats of global warming and the increasing demand for sustainable resources have driven research towards identifying resilient organisms capable of thriving under changing environmental conditions. A recently identified strain of *Neopyropia yezoensis* from Daebudo has demonstrated the ability to grow even under elevated temperatures. Understanding the genetic and molecular mechanisms underlying this resilience is crucial for the development of heat‐tolerant cultivars.

**Methods:**

This study investigates the characteristics of the newly isolated strain *N. yezoensis* (Daebudo) through comprehensive transcriptome analysis on samples cultivated at various temperatures. Transcript reads were aligned to the genome sequences of *N. yezoensis* (susabi‐nori) and *Neoporphyra haitanensis*. Aligned and unaligned reads were assembled separately, generating 45,089 transcripts and 105,750 transcripts, respectively. The transcripts were annotated using homologous sequences from the Swiss‐Prot, NCBI NR, Pfam, SignalP, and KEGG databases. Differentially expressed gene (DEG) analysis was performed, and gene function classification (Gene Ontology) was conducted using BLASTX and BLASTP results from the Swiss‐Prot database.

**Results:**

Some transcripts that were upregulated under higher temperatures were found to be involved in key metabolic pathways such as glycolysis or photosynthesis. Also, several DEGs, including heat shock proteins and elongation factor 1 alpha, were identified as potential contributors to high‐temperature tolerance. These DEGs are likely involved in cellular stress response and protein synthesis, facilitating growth under elevated temperatures.

**Conclusion:**

The findings provide new molecular‐level insights into the growth mechanisms of *N. yezoensis* under heat stress. This information can be applied to the development of new cultivars with enhanced growth and heat tolerance, supporting sustainable aquaculture in the face of climate change.

## 1. Introduction

*Neopyropia* is an economically important marine crop grown in intertidal zones as a popular edible macroalgae. It is widely cultivated in the cold waters of East Asian countries such as China, Japan, and Korea, with an annual harvest of more than 1 million tons [1]. *Neopyropia yezoensis* was first cultivated around the year 1650 in Korea, and since 1990, the production volume has dramatically increased due to the advancement of cultivation techniques and the automation of facilities. [2]. Over the last few decades, as the demand for laver increased, a great deal of effort has been placed in increasing the production rate of *N. yezoensis* cultivation through pursuits like high‐production strain selection [3]. Despite the absence of locomotor capabilities to respond directly to environmental changes, plants employ alternative adaptive mechanisms to adapt to various environmental stresses. Temperature stress, in particular, stands out as one of the most widespread environmental challenges faced by plants [4]. With global warming, the risk, complexity, and uncertainty of operating aquaculture systems increase dramatically [5]. Because *N. yezoensis* is a cold water species that grows in a narrow range of temperatures at around 10°C, if the water temperature increases, the production and quality will drop [6].

For *Neopyropia* aquaculture, some heat‐tolerant strains of *N. yezoensis* have been studied, with various heat‐tolerant mutants of *N. yezoensis* obtained via gamma radiation [7, 8], heat stress [9], or chemical treatment [10]. Although several studies have been conducted to elucidate the molecular mechanisms of these mutants′ heat‐tolerant phenotypes, research into their full mechanisms is still in the early stage [3]. To investigate the molecular mechanism associated with heat‐tolerant mutants, transcriptome analysis using high‐throughput RNA sequencing (RNA‐seq) can be used to identify expressed genes in an assembled transcriptome, offering insights into previously unknown metabolic pathways related to heat tolerance [11].

In this study, we analyzed a newly isolated strain of *N. yezoensis* (Daebudo), which was found on Daebudo island, Ansan, Republic of Korea. It has been confirmed that *N. yezoensis* Daebudo grows well even at a rather high temperature. To find out the specific genes associated with this phenotype, a transcriptome analysis was conducted with the aim of enhancing our understanding of the mechanisms related to heat tolerance in seaweed, contributing valuable insights for further research in this field.

## 2. Materials and Methods

### 2.1. Cultivation of *N. yezoensis* Daebudo and mRNA Sequencing

*N. yezoensis* Daebudo was received from the National Institute of Fisheries Science (Mokpo, Republic of Korea). To analyze gene expression changes associated with temperature differences, cultivation was carried out in triplicate at 5°C, 10°C, 15°C, and 20°C. In this study, *N. yezoensis* blades were cultivated in temperature‐controlled chambers. Several blade fragments were prepared from a single large blade and placed in flasks with aeration at the designated temperatures. mRNA was prepared following previously reported protocols [11] (Figure 1). Triplicate samples at each temperature were obtained, and samples for each temperature are referred to as D5, D10, D15, and D20, respectively, in this paper. The RNA‐seq was performed at Macrogen (Seoul, Republic of Korea).

Figure 1Images of *N. yezoensis* Daebudo cultivated at (a) 5°C, (b) 10°C, (c) 15°C, and (d) 20°C.

**Figure:**
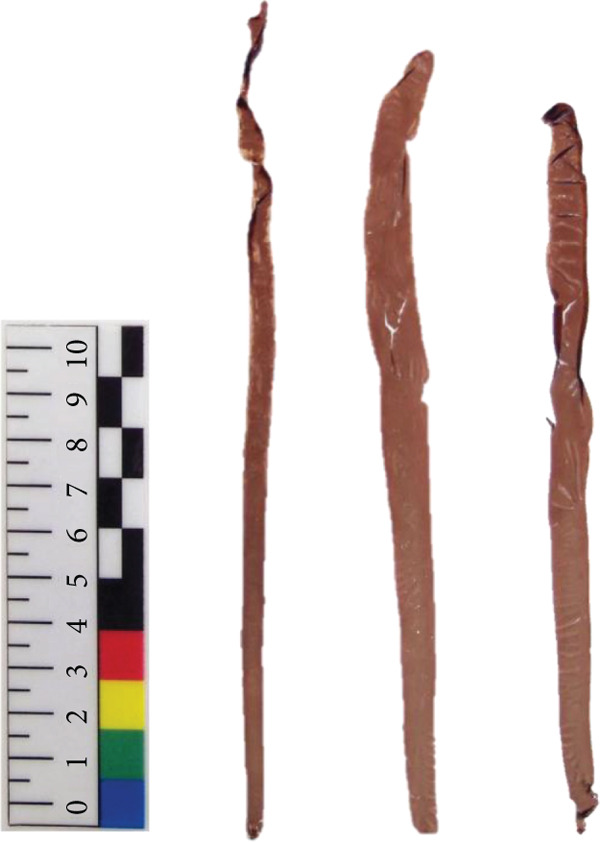
(a)

**Figure:**
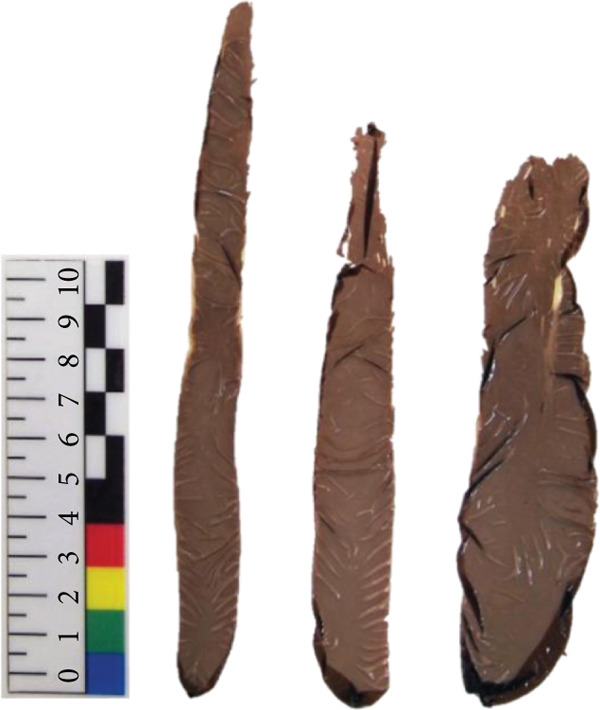
(b)

**Figure:**
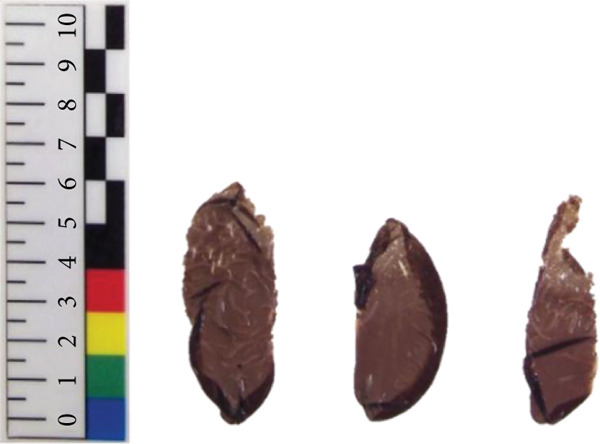
(c)

**Figure:**
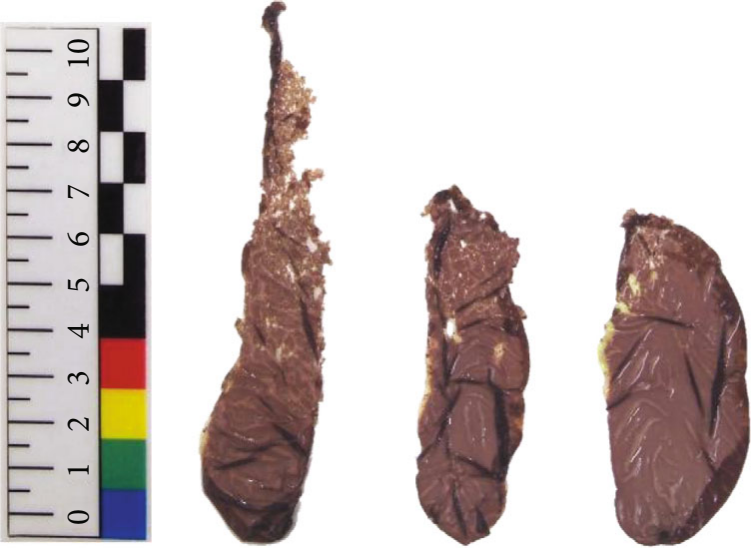
(d)

### 2.2. Data Preprocessing

Then, 24 raw RNA‐seq data files in FASTQ format, corresponding to forward and reverse paired‐end reads from triplicate samples at each temperature treatment, were obtained. To remove Illumina TruSeq Universal Adapter and TruSeq Index Adapters sequences and low‐quality reads, Trimmomatic v0.39 was used for preprocessing with the parameters “ILLUMINACLIP: TruSeq3‐PE.fa: 2:30: 10 LEADING:5 TRAILING:5 SLIDINGWINDOW: 4:5 MINLEN:25,” obtaining the clean read data [12].

The trimmed high‐quality reads were mapped to *N. yezoensis* sequences and *N. haitanensis* sequences to classify aligned reads. Unaligned reads could represent contamination from other species′ sequences and were filtered using the alignment program BBmap/BBsplit [13]. Reads that fail to align to the *N. yezoensis* genome are highly likely to represent contamination. To decontaminate the unaligned contigs, functional annotation tools and taxonomic assignment pipelines were used. Sequences clearly identified as contaminants were excluded from downstream analyses. After removing the contaminants, de novo assembly was applied.

### 2.3. Alignment and Assembly

Since the *N. yezoensis* Daebudo strain′s genome is unknown, de novo assembly for all the reads has high error rates and takes a long time, so alignments using STAR (Spliced Transcripts Alignment to a Reference) [14] were performed first using the genomes of *N. yezoensis* (susabi‐nori) and *N. haitanensis*, the two most common *Neopyropia* species in South Korea [15]. Details of the genomes downloaded from NCBI are provided in Table 1. The aligned reads were kept as mapped reads and unaligned reads as unmapped reads. Then, the mapped reads were assembled using the genome‐guided assembly method (reference‐based assembly) [16], and unmapped reads were assembled using the de novo assembly method [17]. Both were performed using the short‐read assembling program Trinity v2.14.0 with the default parameters [18]. In the case of de novo assembly, the sequence of the contaminant can be included, so the decontamination process is required to remove them. To filter out other species, assembled contigs were annotated by DIAMOND BLASTP and BLASTN using the NCBI nonredundant (NR) database to collect taxonomic information. Decontaminated unmapped reads were then de novo assembled again. Then, ORF prediction and redundant protein removal were performed using the same methods as with the genome‐guided assembly.

**Table 1:** Genomic sequences from the NCBI database that were used in this study.

**Taxon**	**Assembly**	**Chromosome**	**Size (bp)**	**GC content (%)**
*Neopyropia yezoensis (susabi-nori)*	GCA_009829735.1	CM020618.1	43,592,136	65
		CM020619.1	34,332,347	65
		CM020620.1	29,394,270	64.5

*Neoporphyra haitanensis*	GCA_008729055.1		53.2546 M	67.8

*Lawsonella clevelandensis*	GCF_001293125.1		1.9 M	58.5

*Cutibacterium acnes* strain JCM	GCF_00376705.1		2.49 M	60.1

To evaluate whether the assembly was performed well, the reads designated as the input file for the assembly were mapped back to the assembly result file; that is, with the Trinity result file as a reference, using the reference mapping software Bowtie 2 v2.4.5 [19].

Transcript abundance was estimated by employing the Trinity transcript quantification workflow based on Salmon′s wicked‐fast quantification method. The N50 value, the shortest contig for which longer and equal‐length contigs cover at least 50% of the assembly, was used to assess the quality of our data.

TransDecoder was used to identify the coding region within transcript sequences, including both de novo and genome‐guided RNA‐seq transcript assemblies. CD‐HIT‐EST was used to get a nonredundancy dataset by clustering sequences based on similarity. The ORF peptide sequences predicted by TransDecoder were clustered with CD‐HIT into 100% identity groups.

### 2.4. Functional Annotation of Transcripts

Functional annotation was performed to understand the functions of the assembled transcripts using the Trinotate workflow. We performed BLASTP and BLASTX homology searches against the Swiss‐Prot database. An SQLite database annotated with information from BLAST search results, signalP, and tmhmm is used as the input for Trinotate. The peptide sequences of the candidate ORFs in the TransDecoder result file were used to perform a homology search against the NCBI NR and Swiss‐Prot databases. The NCBI NR database is too large and time‐consuming to use with the standard BLAST+ program, so we used the DIAMOND BLASTP aligner to search this database. The results of these searches were used to identify taxonomic content, such as protein names, and organism information through the uniprot‐id mapping. Pathway annotations were applied using the Kyoto Encyclopedia of Genes and Genomes (KEGG) IDs applied in the Trinotate output.

### 2.5. Differentially Expressed Gene Analysis

After all the previous analyses, DEG analysis was performed to identify how gene expression varied under different temperatures with the aim of furthering our understanding high‐temperature tolerance‐related genes [20]. The DEG analysis was performed using the DESeq2 method following the Trinity workflow with the Salmon result file as the input. To properly control the number of DEGs, a *p* value cutoff of ≤ 0.005 and a log_2_ foldchange ratio of ≥ 3 was used for mapped‐genes and a log_2_ foldchange ratio of ≥ 4 was used for unmapped genes.

## 3. Results and Discussion

### 3.1. Cultivation and RNA‐seq of *N. yezoensis* Daebudo

*N. yezoensis* Daebudo was cultivated in triplicate at 5°C, 10°C, 15°C, and 20°C to see if there were any differences in growth. *N. yezoensis* is a red alga cultivated in cold coastal waters of East Asia, with optimal growth typically occurring around 10°C. Previous studies have shown that this species grows stably within 5°C–15°C, while growth declines at 20°C or higher due to high‐temperature stress, which can reduce pigment levels, nitrogen uptake, and growth rates. Based on this information and prior studies that used similar temperature ranges, we selected 5°C, 10°C, 15°C, and 20°C for our experiments.

Previous study which investigated the impact of temperature on the relative growth rate of *N. yezoensis* revealed a decrease in growth rate with increasing temperature. At 13°C, the growth rate was relatively maintained, whereas at higher temperatures such as 17°C, 21°C, and 23°C, the growth was severely damaged [21]. However, in the case of *N. yezoensis* Daebudo grown at 15°C, the length was shorter than that grown at 10°C, but the width was widened. Especially, *N. yezoensis* Daebudo did not show any difference in growth even under conditions of 20°C compared to that of 15°C (Figure 1). The average biomass of a blade were 4.22 ± 1.65 mg at 5°C, 11.47 ± 2.7 mg at 10°C, 3.94 ± 1.67 mg at 15°C, and 4.64 ± 1.34 mg at 20°C, respectively. It is therefore worth discussing that this species has high‐temperature tolerance.

### 3.2. Data Processing

After trimming to remove adapter sequences and low‐quality reads, an average of 89.21%, 87.29%, 89.31%, and 89.66% of the raw reads remained in the D5, D10, D15, and D20 temperature treatments, respectively (Table 2). Also, the GC% of the clean data for most samples was 64%, while it was 63% for the D5_2 sample and 65% for all the D20 samples.

**Table 2:** Read statistics, including raw and clean read data.

**Samples**		**Raw read**			**Clean reads**			
		**Total sequences**	**Sequence length**	**GC content**	**Total sequences**	**Sequence length**	**GC content**	**% of raw read**
D5	1	13,094,290	151	64%	11,501,724	150–151	64%	87.84%
	2	13,140,457	151	63%	11,791,100	150–151	63%	89.73%
	3	13,622,298	151	63%	12,268,149	150–151	64%	90.06%

D10	1	15,609,781	151	64%	13,557,710	150–151	64%	86.85%
	2	16,578,662	151	64%	14,426,990	150–151	64%	87.02%
	3	12,507,309	151	64%	11,006,283	150–151	64%	88%

D15	1	12,669,125	151	64%	11,290,563	150–151	64%	89.12%
	2	12,288,070	151	64%	11,131,767	150–151	64%	90.59%
	3	17,143,689	151	64%	15,124,091	40–151	64%	88.22%

D20	1	13,356,831	151	65%	11,977,149	150–151	65%	89.67%
	2	15,884,146	151	65%	14,201,063	150–151	65%	89.4%
	3	15,964,882	151	64%	14,353,800	150–151	65%	89.91%

Trimmed data were aligned to *N. yezoensis* (susabi‐nori) and *N. haitanensis* genome sequences. On average, 81.43%, 83.43%, 80.82%, and 88.58% of reads were mapped to the reference genome at 5°C, 10°C, 15°C, and 20°C, respectively. Of these, the reads mapped to *N. yezoensis* were 81.52%, 83.44%, 80.88%, and 88.57%, and the reads mapped to *N. haitanensis* were 7.27%, 5.65%, 5.94%, and 7.03% by temperature, respectively. Most of the reads were mapped to *N. yezoensis* (susabi‐nori), and a somewhat lower mapping rate can be seen for *N. haitanensis* (Table 3). Therefore, this sample was named *N. yezoensis* Daebudo. Mapping to both *N. yezoensis* and *N. haitanensis* genomes was not intended for species identification. Although we observed ~80% sequence similarity with *N. yezoensis*, our primary objective was to achieve a more accurate and comprehensive transcriptome assembly. Relying solely on de novo assembly for all reads would have been more error‐prone and computationally demanding. The genomes of these two species—both common in the Republic of Korea—were used to improve alignment accuracy, reduce annotation bias, and maximize transcript recovery. This approach could also help account for the possibility that our newly isolated strain may possess divergent or mixed genomic features.

**Table 3:** Statistics of the reads aligned to the genomes of *N. yezoensis* and *N. haitanensis* (STAR).

		**Number of input reads**	**Uniquely mapped reads (number)**	**Uniquely mapped reads (%)**	**Average mapped length**
5	1	11,501,724	9,776,388	85.00%	299.17
	2	11,791,100	9,194,524	77.98%	299.05
	3	12,268,149	9,976,201	81.32%	299.11

10	1	13,557,710	11,293,840	83.30%	299.17
	2	14,426,990	12,037,315	83.44%	299.27
	3	11,006,283	9,196,022	83.55%	299.29

15	1	11,290,563	9,096,059	80.56%	299.2
	2	11,131,767	9,016,415	81.00%	299.02
	3	15,124,091	12,237,650	80.91%	298.9

20	1	11,977,149	10,683,207	89.20%	299.02
	2	14,201,063	12,559,921	88.44%	299.01
	3	14,353,800	12,644,331	88.09%	299.02

The aligned reads in each sample were assembled using Trinity’s genome‐guided assembly mode. A total of 45,781 transcripts were thus obtained after redundancy removal (Table 4). The average GC content in these transcripts was 69.93%, while the GC content of the raw and clean reads was 63%–65%. The average and median contig lengths were 702.41 and 507, respectively, while the contig N50 value was 780, indicating that the assembly was done well. In general, ExN50 is widely used in de novo transcriptome assemblies to select biologically meaningful transcripts based on expression coverage. However, in our case, most contigs were assembled via genome‐guided methods. Because ExN50 tends to favor highly expressed transcripts, we chose CD‐HIT‐EST and CD‐HIT to retain low‐abundance transcripts that may still be biologically important. While ExN50 can effectively reduce assembly noise, our priority in this study was to avoid losing rare transcripts potentially relevant to heat tolerance.

**Table 4:** Statistics based on assembled transcript contigs in the mapped and unmapped data after removing redundant predicted proteins.

	**Mapped**	**Unmapped**
Total Trinity genes	23,516	87,232
Total Trinity transcripts	45,781	106,281
GC content (%)	69.93%	60.77%
Contig N10	2553	2422
Contig N50	780	680
Median contig length	507	315
Average contig	702.41	522.60
Total assembled bases	32,156,910	55,542,775

The unaligned reads (roughly 17%) for each sample were assembled using the de novo assembly mode. Taxonomic information for each NR_id was organized, and the Top 2 bacteria sequences, *Lawsonella clevelandensis* and *Cutibacterium acnes* strain JCM, were selected. Putative contaminant sequences closely related to these genomes of *L. clevelandensis* and *C. acnes* were removed from the unmapped read file.

For the assembled groups from unmapped reads, GC content was 64.51%, like those of the raw and clean reads, 63%–65%. The average and median contig lengths were 598.39 and 465, respectively, and the N50 value was 630, again indicating that the assembly was done well.

To further confirm that the assembly reflected the reads well, the reads used as the input file were mapped to the resulting assembly file with Bowtie 2 v2.4.5 (Table 5). The average mapping rate of mapped reads was 97.48%, 97.67%, 95.13%, and 97.97% for the D5, D10, D15, and D20 treatments, respectively, while the average mapping rate of unmapped reads was 90.24%, 87.45%, 88.96% and 84.48%, respectively. Since de novo assembly was analyzed using only read redundancy, the mapping rate is a little lower than that of mapped reads. All the assembled transcripts were annotated using Trinotate. This annotation information was combined with a transcript determined as DEG to provide information on which metabolic pathway DEGs play a role. The results of functional annotation were shown in Table 6.

**Table 5:** Summary of the mapping rates with Bowtie 2.

**Samples**		**Mapped**	**Unmapped**
D5	1	98.52%	91.11%
	2	96.56%	93.21%
	3	97.37%	92.74%

D10	1	97.06%	89.38%
	2	96.83%	88.36%
	3	97.02%	90.36%

D15	1	95.23%	90.53%
	2	95.01%	90.44%
	3	95.16%	91.04%

D20	1	98.32%	89.66%
	2	97.95%	85.91%
	3	97.65%	79.84%

**Table 6:** Summary of the functional annotation results.

	**Mapped**		**Unmapped**	
	**Number of transcripts**	**% of total transcripts**	**Number of transcripts**	**% of total transcripts**
Annotated in Swiss‐Prot (BLASTX)	12,583	24.21%	32,592	29.85%
Annotated in Swiss‐Prot (BLASTP)	10,477	20.16%	18,956	17.36%
Annotated in NCBI NR	17,404	33.49%	27,136	24.85%
Annotated in Pfam	11,091	21.34%	19,339	17.71%
Annotated in KEGG	12,479	24.01%	28,095	25.73%

### 3.3. Differentially Expressed Gene Analysis

With thresholds of a *p* value cutoff of ≤ 0.05 and a log_2_ fold change ratio of ≥ 2, DEGs were analyzed. The D5 assembly was compared to the D10, D15, and D20 assemblies, identifying 1756, 1601, and 1605 upregulated genes and 1495, 1304, and 1423 downregulated genes in the D10, D15, and D20 assemblies, respectively. Overall, 860 genes were upregulated and 895 genes were downregulated in all three treatments. Due to extensive number of DEGs, DEG analysis with more stringent thresholds (a *p* value ≤ 0.005 and log2 fold change ≥ 3) was carried out. The D5 assembly was compared to the D10, D15, and D20 assemblies, identifying 956, 938, and 889 upregulated genes and 360, 303, and 436 downregulated genes in the D10, D15, and D20 assemblies, respectively. Overall, 328 genes were upregulated and 129 genes were downregulated in all three treatments (Figure 2a,b). In the pathway analysis, 311 of the 1316 DEGs in D10, 291 of the 1241 DEGs in D15, and 330 of the 1325 DEGs in D20 were annotated to KEGG Orthology IDs, resulting in 307, 293, and 305 pathways in D10, D15 and D20, respectively, identified with KEGG Mapper search. “Metabolic pathway”–related genes were the most common DEGs for all samples, followed by “biosynthesis of secondary metabolites” and then “microbial metabolism in diverse environments.” Figure 3a shows the most matched pathways following these three among the D10, D15, and D20 treatments.

Figure 2Venn diagrams of DEGs, including the number of (a) upregulated and (b) downregulated differentially expressed genes (DEGs) in the mapped reads of the D10 (10°C), D15 (15°C), and D20 (20°C) treatment groups compared to the D5 (5°C) group. The (c) upregulated and (d) downregulated DEGs in the unmapped read of the D10, D15, and D20 treatment groups compared to the D5 group.

**Figure:**
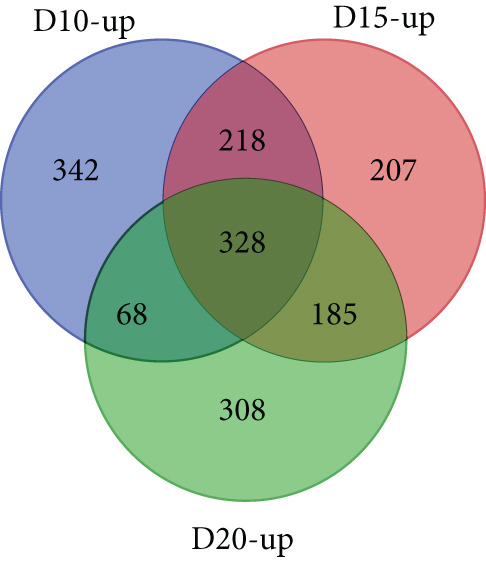
(a)

**Figure:**
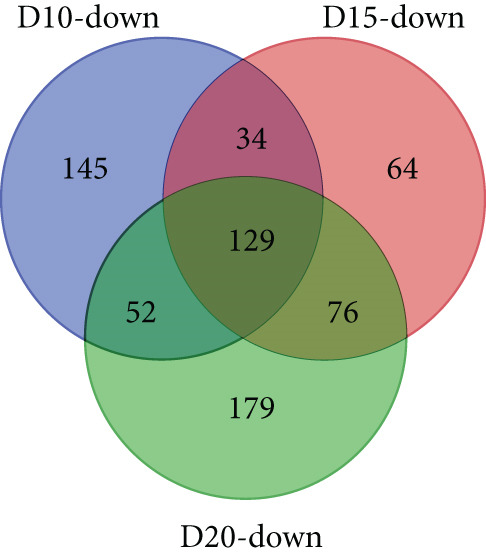
(b)

**Figure:**
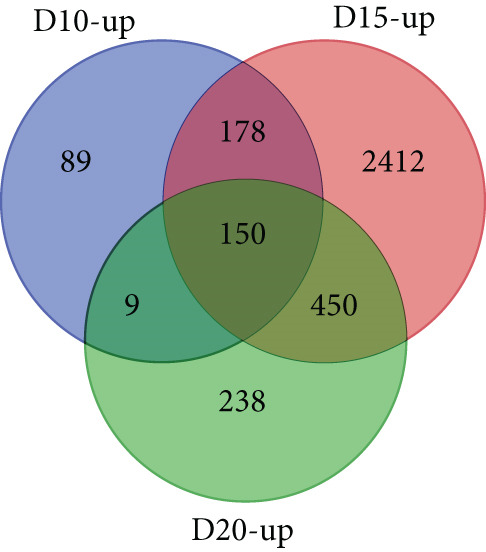
(c)

**Figure:**
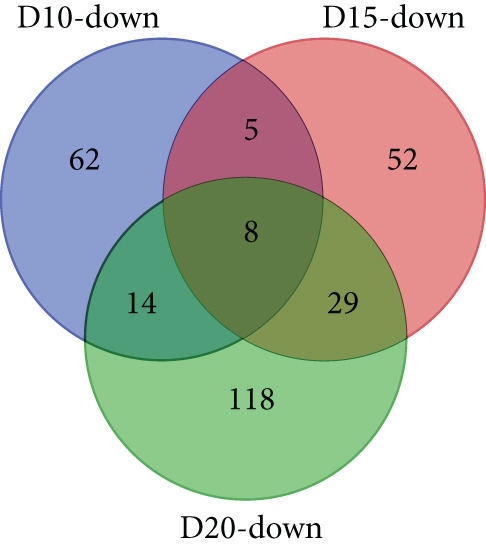
(d)

Figure 3The most abundant KEGG pathways annotated to the (a) mapped and (b) unmapped reads in the D10 (white bars), D15 (gray bars), and D20 (dark‐gray bars) treatment groups’ data, as compared with the D5 data. Ribosome‐related pathways, such as “Ribosome” and “Ribosome biogenesis in eukaryotes,” have high counts in all data, especially in D20.

**Figure:**
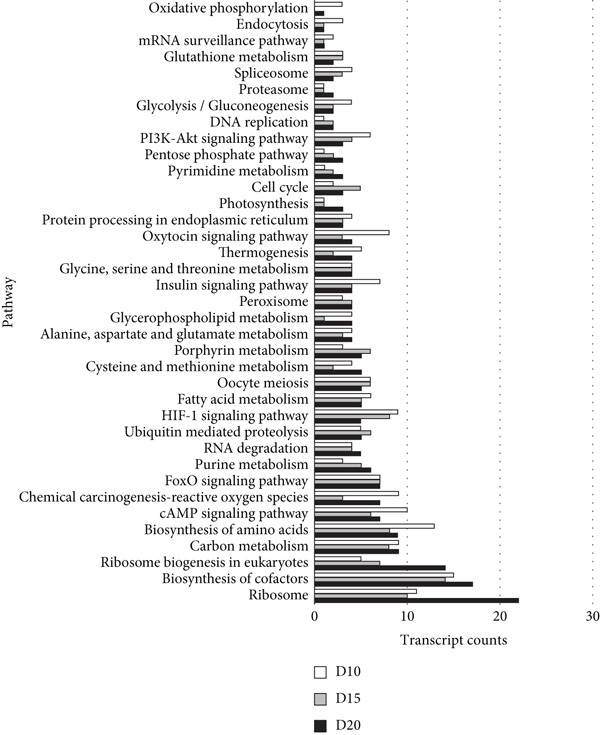
(a)

**Figure:**
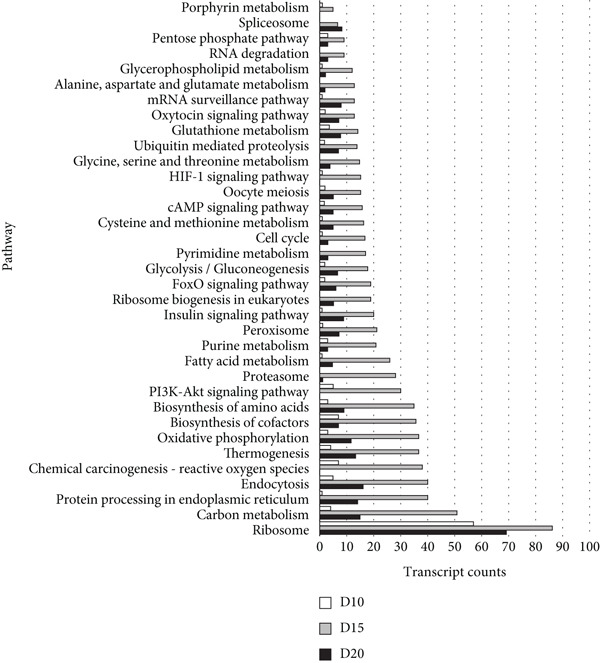
(b)

From the unmapped reads, we identified 426, 3190, and 847 upregulated genes and 89, 94, and 169 downregulated genes in the D10, D15, and D20 assemblies, respectively. Of these, 150 genes were upregulated and eight genes were downregulated in all three assemblies (Figure 2c,d). In the KEGG analysis, 194 of the 515 DEGs in D10, 1597 of the 3284 DEGs in D15, and 455 of the 1016 DEGs in D20 were annotated, resulting in 212, 392, and 304 identified pathways, in the D10, D15, and D20 treatments, respectively. Metabolic pathway–related genes were the most common DEGs in treatments D15 and D20, while “Ribosome” was the most matched pathway in D10. The most abundant KEGG pathways in unmapped genes following “metabolic pathway” are presented in Figure 3b. While *N. yezoensis* generally exhibits optimal growth around 10°C, our objective was to identify genes associated with heat tolerance in a newly isolated strain that also grows well at higher temperatures. To capture temperature‐dependent expression trends, we selected 5°C as the baseline for comparison. This lower temperature reference made it easier to detect the progressive upregulation of heat‐responsive genes as temperatures increased from 10°C to 20°C. In contrast, using 10°C as the reference would have reduced the ability to observe these gradual changes. In fact, we also compared the D10 samples with the D15 and D20 samples; however, the differential expression analysis produced less distinct patterns.

**Figure 4:**
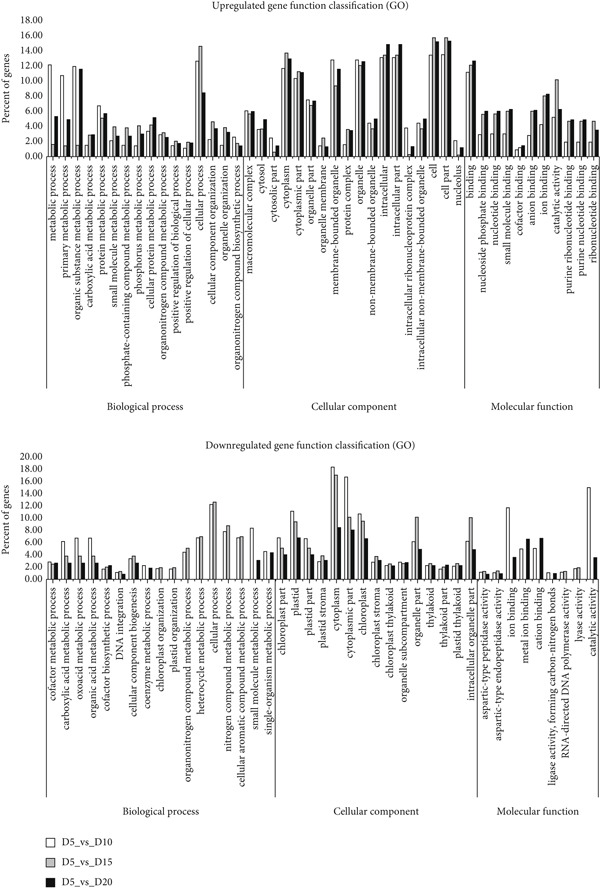
Gene function classification based on a Gene Ontology (GO) analysis of the DEGs in the D10 (white bars), D15 (gray bars), and D20 (lined bars) treatment groups’ data. The GO functional IDs are arranged by category along the *x*‐axis.

We performed a Gene Ontology (GO) enrichment analysis to find out how many DEGs exist in a particular pathway using the DAVID (Database for Annotation, Visualization and Integrated Discovery) online resource. The GO enrichment analysis is performed using the BLASTX and BLASTP search results from the Swiss‐Prot database. Among the 1382, 4128, and 1732 upregulated genes, 608, 2563, and 993 DEGs (including mapped and unmapped genes), respectively, were annotated, and 180, 159, and 228 DEGs in 449, 397, and 605 downregulated genes were annotated in both BLAST searches. *N. yezoensis* (susabi‐nori), which had a pretty high mapping ratio with our Daebudo strain reads, is not a model species, so the false discovery rate value (*p* ≤ 0.005) was excluded from the GO analysis. These annotated genes used to get the percentage values for the DEGs in Figure 4. The “intracellular” annotation in the cellular component (CC) category and “binding” in the molecular function (MF) category were noticeably enriched in the upregulated transcripts, while the “chloroplast” annotation in CC and “catalytic activity” in MF were noticeably enriched in the downregulated transcripts. Additionally, the “cellular process” annotation in the biological process (BP) category and “cytoplasm” in CC were enriched in both up‐ and downregulated transcripts.

### 3.4. Gene Candidates Related to Heat Tolerance

We integrated mapped and unmapped data to compile a list of candidate genes in *N. yezoensis* Daebudo seaweed grown in a somewhat high‐temperature environment. For DEG selection, we used 5°C as the reference temperature and identified genes whose expression levels increased at 10°C, 15°C, and 20°C, respectively. We further filtered these genes to include only those showing a consistent upregulation trend across increasing temperatures. Expression levels of the important genes involved were found to increase with temperature (Table 7). In total, there were 20 upregulated and 14 downregulated genes. Various heat‐resistant proteins such as “heat shock protein (HSP) 70,” “17.5‐kDa class I HSP,” and “HSS1” were observed. Studies have also shown that “elongation factor 1‐alpha (EF1A)” can affect a heat‐tolerance phenotype in *N. yezoensis*, and in Daebudo, the expression of *EF1A* increased with increasing temperatures. The “ATP synthase subunit beta” and “eukaryotic translational initiation factor 5A” proteins also affected the growth rate of the Daebudo seaweed strain. We note that the “ABC transporter family” and “molybdopterin synthase” coexisted in mapped and unmapped upregulated transcripts and that “tubulin‐folding cofactor C” coexisted in mapped and unmapped downregulated transcripts.

**Table 7:** List of candidate genes identified in *Neopyropia yezoensis* (Daebudo) cultivated at high temperatures.

	**Trinity_ID**	**Description**	**E** **value**
mapped_up	TRINITY_GG_28_c446_g1_i3	Multidrug and toxin extrusion protein 1	*E* : 6.28e − 10
	TRINITY_GG_214_c588_g1_i1	Acyl‐coenzyme A oxidase 2, peroxisomal	*E* : 1.39e − 26
	TRINITY_GG_28_c512_g1_i6	17.5 kDa class I heat shock protein	*E* : 2.07e − 07
	TRINITY_GG_292_c0_g1_i1	Heat shock 70 kDa protein	*E* : 3.24e − 59
	TRINITY_GG_28_c5869_g1_i1	RNA‐binding protein NOB1	*E* : 4.6e − 21
	TRINITY_GG_214_c347_g1_i4	Protein antagonist of like heterochromatin protein 1	*E* : 1.37e − 09
	TRINITY_GG_28_c162_g1_i5	Elongation factor 1‐alpha	*E* : 0
	TRINITY_GG_302_c105_g1_i1	GTP‐binding nuclear protein Ran	*E* : 1.46e − 65
	TRINITY_GG_302_c726_g1_i1	Putative ribosome biogenesis protein C8F11.04	*E* : 1.11e − 36
	TRINITY_GG_214_c758_g1_i1	Probable dynein light chain	*E* : 3e − 18
	TRINITY_GG_302_c308_g6_i1	Germin‐like protein subfamily 1 member 6	*E* : 1.01e − 14
	TRINITY_GG_214_c418_g1_i1	ABC transporter I family member 10	*E* : 3.65e − 09
	TRINITY_GG_302_c15_g1_i7	Heat shock protein HSS1	*E* : 0
	TRINITY_GG_28_c43_g1_i15	Molybdopterin synthase catalytic subunit 1	*E* : 6.04e − 08
	TRINITY_GG_28_c11_g1_i13	Fructose‐bisphosphate aldolase	*E* : 1.27e − 98
	TRINITY_GG_214_c89_g1_i14	Germin‐like protein 5‐1	*E* : 4.69e − 13

unmapped_up	TRINITY_DN3374_c0_g1_i4	Molybdopterin synthase catalytic subunit 2	*E* : 5.78e − 29
	TRINITY_DN160_c0_g1_i1	Actin	*E* : 8.59e − 154
	TRINITY_DN1280_c0_g1_i1	ABC transporter C family member 8	*E* : 1.93e − 21
	TRINITY_DN53275_c0_g1_i1	Actin‐related protein 2/3 complex subunit 3	*E* : 3.96e − 51

mapped_down	TRINITY_GG_302_c4050_g1_i1	Histone H3.3C	*E* : 1.74e − 16
	TRINITY_GG_214_c194_g1_i2	Disulfide‐bond oxidoreductase YfcG	*E* : 1.74e − 34
	TRINITY_GG_214_c665_g1_i2	Protein sym‐1	*E* : 1.03e − 23
	TRINITY_GG_302_c7192_g1_i1	Estradiol 17‐beta‐dehydrogenase 1	*E* : 1.38e − 38
	TRINITY_GG_28_c309_g2_i1	Tubulin‐folding cofactor C	*E* : 3.46e − 18
	TRINITY_GG_28_c86_g5_i1	Retinol dehydrogenase 8	*E* : 2.32e − 22
	TRINITY_GG_28_c821_g2_i6	Carbonic anhydrase	*E* : 7.03e − 18
	TRINITY_GG_214_c175_g1_i11	TyrosinetRNA ligase	*E* : 3.71e − 110
	TRINITY_GG_302_c2499_g1_i1	Probable aspartate–semialdehyde dehydrogenase	*E* : 1.09e − 101
	TRINITY_GG_214_c856_g1_i17	Asparagine synthetase (glutamine‐hydrolyzing) 2	*E* : 5.76e − 113
	TRINITY_GG_144_c13_g1_i1	Sulfite reductase (ferredoxin)	*E* : 3.81e − 28
	TRINITY_GG_28_c706_g1_i3	Bifunctional polynucleotide phosphatase/kinase	*E* : 6.28e − 10

Unmapped_down	TRINITY_DN2166_c0_g4_i1	Tubulin‐folding cofactor C	*E* : 1.48e − 17
	TRINITY_DN744_c0_g1_i4	Ferredoxin‐‐NADP reductase	*E* : 2.1e − 131

Because the optimal growth temperature of *N. yezoensis* is around 10°C, a comparison by temperature was conducted based on the D10 treatment group. First, the Histone H3 coding gene had the highest expression difference when comparing D10 to D15 and D10 to D20. Germin‐like protein, which is known to be involved in plant responses to abiotic and biotic stressors, was found to have a high expression change when comparing D10 and D15. Fructose‐bisphosphate aldolase is a cytosolic enzyme that activates a step in glycolysis, a basic metabolic pathway in the ATP synthesis process, so this upregulated expression can greatly affect cell growth [22].

HSPs are universal proteins synthesized in all plant and animal species that have been studied in cells that are briefly exposed to temperatures higher than their optimal growth temperature. Since they can be induced by other stressors, such as oxidants, toxins, heavy metals, and free radicals, HSPs are called the “stress protein.” They are classified as HSP100, HSP90, HSP70, HSP60, and small HSP families according to their molecular masses. Their response to heat has a different mechanism than the usual “response to stimulation,” which immediately changes the state of the cell. The N‐terminal nucleotide binding domain (NTD) ligates to the C‐terminal substrate binding domain through a flexible linker. In its ADP‐bound state, HSP70 recognizes the substrate at the SBD domain which consists of two subdomains, SBD_*α*_ and SBD_*β*_. The nuclear exchange factors (NEFs) mediate the exchange from ADP to ATP, leading to the rotation of HSP70 and the covering of the NBD by the SBD*α* helix lid when ATP is bound. In the ATP‐bound state, HSP70 exhibits low affinity for the substrate. Interaction with the cochaperone J‐domain protein triggers ATP hydrolysis in HSP70, causing it to revert to the ADP‐bound state and initiating another cycle of the process [23]. Protein biosynthesis in the cell is suppressed when heat stress occurs, and HSP gene expression is then promptly enhanced. After the response to heat shock, the process is attenuated and the cell returns to its normal state [24]. Molecular chaperones, including heat shock proteins, are produced in response to stressful conditions such as heat, UV light, and even cold. In this study, “7.5‐kDa class I HSP,” “HSP70,” and “HSP HSS1” were observed. The expression of *HSP 70* and *HSP HSS1* increased as the temperature increased, while “7.5‐kDa class I HSP” was found to have slightly lower expression at 20°C than at 15°C but still significantly higher expression than at 10°C. These regular changes in HSPs can affect a phenotype that grows well, even at somewhat high temperatures.

The enzyme, ATP synthase, is the key to cell respiration along with photosystem I, photosystem II, and cytochrome *b*_6_ *f*. It is composed of two rotary motors/generators, *F*_0_ and *F*_1_: *F*_0_ plays the role of proton driver and fixes the ATP synthase to the inner membrane, while *F*_1_ functions in ATP synthesis. The *F*_1_ region has *α* and *β* subunits, which are arranged alternately to form a pseudohexagonal structure called (*αβ*)_3_. In the center, a *γ*‐subunit forms the central axis. The three *β* subunits, *β*_E_, *β*_DP_, and *β*_TP_, are empty, loaded with MgADP and loaded with a nonhydrolyzable ATP analog, respectively. The *β*_E_ subunit binds a new ATP molecule, then *β*_DP,_ with its helix‐turn‐helix motif (hinge), empties the respective nucleotide‐binding site, and finally, at *β*_TP_ site, ATP is split into bound ADP and P_i_ [25]. The ATP molecule is the organic compound that supplies energy to carry out many life activities in living cells. The expression of transcripts identified as “ATP synthase beta subunit” increased from 10°C to 15°C, suggesting that the “ATP synthase beta subunit” might be responsible for the heat‐tolerance phenotype of *N. yezoensis* Daebudo.

The eukaryotic translational initiation factor 5A (eIF5A) protein, which has been found in all eukaryotic organisms, has an inactive precursor that is activated by a posttranslational modification mechanism. Hypusine (Hpu) is a derivative of lysine (Lys) that is formed by posttranslational modification of a Lys residue in eIF5A [26]. This modification is performed in a two‐step reaction. First, Lys is converted to deoxyhypusine by adding a butylamine group from spermidine. Then, deoxyhypusine hydroxylase converts deoxyhypusine into Hpu. Interactions between the putative translation initiation factor and the ribosomal protein L5, as well as the translating 80S ribosomal complex, provide evidence for the involvement of eIF5A in protein translation. The hypusination of eIF5A is crucial for the survival of yeast, and Hpu‐containing eIF5A might be involved in the control of cell proliferation and apoptosis [27]. A recent study of eIF5A suggests a significant role for eIF5A in a range of physiological processes, such as development and metabolic adaptation, in addition to its well‐known function in facilitating protein translation. It is believed that eIF5A can contribute to posttranscriptional gene regulation by stabilizing ribosomal preinitiation complexes around the start codon. In *N. yezoensis* Daebudo, the expression of eIF5A increased with temperature up to 15°C, but slightly decreased from 15°C to 20°C. Translation is one of the most important factors for life and is the central dogma, so the eIF5A protein′s importance in regulating translation would be extremely broad.

**Figure 5:**
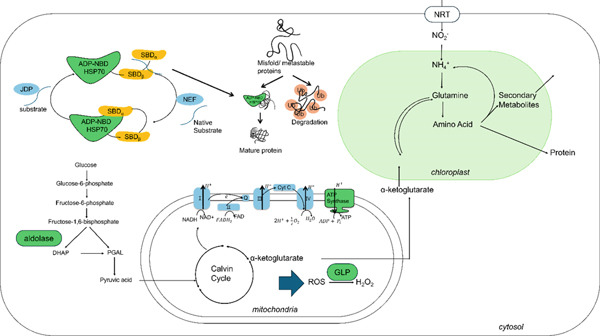
Description of differentially expressed genes (DEGs) related to the high growth rate of the *N. yezoensis* Daebudo.

The germin‐like protein was discovered in germinating wheat grains, but now, it is known to be expressed in a range of plant species. Molecular investigation during wheat germination revealed “germin” that is resistant to proteases. The germin‐like protein is structurally and sequentially similar to germin but does not have the activity of oxalate oxidase, as germin does, so it was called “germin‐like protein (GLP).” The GLPs are a group of developmentally regulated glycoproteins that are known for their beta‐barrel core structure, signal peptide, and association with the cell wall [28]. A recent study found that GLPs have a function in the biotic and abiotic stress responses of different plants. Furthermore, Das et al. (2019) found that the transcription factor binding sites in its promoter regions might be related to genes associated with biotic and abiotic stress tolerance in rice. During specific periods of plant growth and development, the GLPs are differentially expressed to perform various functions. Some studies looking at fungal pathogenesis in cereals have suggested that links between GLPs and QTLs lead to disease and stress resistance, protecting plants from various environmental stressors. The upregulated expression of germin‐like protein may affect the heat stress tolerance of *N. yezoensis* Daebudo. This protein is originally plant‐derived, identified in plants like wheat and rice, but as the germin‐like protein appears to be ubiquitous in plants, it is expected to provide tolerance to abiotic stress in *N.yezoensis* Daebudo as well. These DEGs activities are shown in Figure 5.

## 4. Conclusion

This study was aimed at identifying the related genes with the mechanisms underlying the phenotype of high‐growth rate of *N. yezoensis* Daebudo. To approach this, RNA‐seq of *N. yezoensis* Daebudo grown in different temperature conditions was genome‐guided de novo assembled using *N. haitanensis* and *N. yezoensis* genome as references. Then, we performed the comparative transcriptome analysis with assembled transcripts. In total, 34 transcripts were differentially expressed in *N. yezoensis* Daebudo cultivated at high temperatures. These transcripts are candidates for the high growth rate of *N. yezoensis* Daebudo, associated with different functional groups. Some upregulated transcripts were involved in metabolic pathways like glycolysis or photosynthesis. Heat shock protein, the elongation factor 1‐alpha (EF1A), and germin‐like protein were also identified by DEG analysis.

In fact, it will be possible to confirm the function of candidate genes through experiments that confirm traits by introducing genes into eukaryotic organisms. Additional research concentrating on these candidates, particularly on the genes that have not been characterized before, could unveil new molecular mechanisms responsible for the valuable feature.

## Ethics Statement

The authors have nothing to report.

## Consent

The authors have nothing to report.

## Disclosure

All authors have read and agreed to the final version of the manuscript.

## Conflicts of Interest

The authors declare no conflicts of interest.

## Author Contributions

Jiae Kim conducted the conception and methodology parts and wrote the original draft of the manuscript. Jong‐il Choi reviewed it and made revisions.

## Funding

The research was supported by the Chonnam National University Supporting Program and by the “Regional Innovation System & Education (RISE)” through the Gwangju RISE Center funded by the Ministry of Education (MOE) and the Gwangju Metropolitan Government (2025‐RISE‐05‐011).

## Data Availability

The data used in this study is available from the corresponding author upon request.
